# Exploiting expression patterns across multiple tissues to map expression quantitative trait loci

**DOI:** 10.1186/s12859-016-1123-5

**Published:** 2016-06-24

**Authors:** Chaitanya R. Acharya, Janice M. McCarthy, Kouros Owzar, Andrew S. Allen

**Affiliations:** Program in Computational Biology and Bioinformatics, Duke University, 101 Science Dr, Durham, 27708 USA; Department of Biostatistics and Bioinformatics, Duke University, 2424 Erwin Rd, Durham, 27708 USA

**Keywords:** eQTL mapping, Multiple tissues, Score test, Tissue-specificity

## Abstract

**Background:**

In order to better understand complex diseases, it is important to understand how genetic variation in the regulatory regions affects gene expression. Genetic variants found in these regulatory regions have been shown to activate transcription in a tissue-specific manner. Therefore, it is important to map the aforementioned expression quantitative trait loci (eQTL) using a statistically disciplined approach that jointly models all the tissues and makes use of all the information available to maximize the power of eQTL mapping. In this context, we are proposing a score test-based approach where we model tissue-specificity as a random effect and investigate an overall shift in the gene expression combined with tissue-specific effects due to genetic variants.

**Results:**

Our approach has 1) a distinct computational edge, and 2) comparable performance in terms of statistical power over other currently existing joint modeling approaches such as MetaTissue eQTL and eQTL-BMA. Using simulations, we show that our method increases the power to detect eQTLs when compared to a tissue-by-tissue approach and can exceed the performance, in terms of computational speed, of MetaTissue eQTL and eQTL-BMA. We apply our method to two publicly available expression datasets from normal human brains, one comprised of four brain regions from 150 neuropathologically normal samples and another comprised of ten brain regions from 134 neuropathologically normal samples, and show that by using our method and jointly analyzing multiple brain regions, we identify eQTLs within more genes when compared to three often used existing methods.

**Conclusions:**

Since we employ a score test-based approach, there is no need for parameter estimation under the alternative hypothesis. As a result, model parameters only have to be estimated once per genome, significantly decreasing computation time. Our method also accommodates the analysis of next- generation sequencing data. As an example, by modeling gene transcripts in an analogous fashion to tissues in our current formulation one would be able to test for both a variant overall effect across all isoforms of a gene as well as transcript-specific effects. We implement our approach within the R package JAGUAR, which is now available at the Comprehensive R Archive Network repository.

**Electronic supplementary material:**

The online version of this article (doi:10.1186/s12859-016-1123-5) contains supplementary material, which is available to authorized users.

## Background

**Fig. 1:**
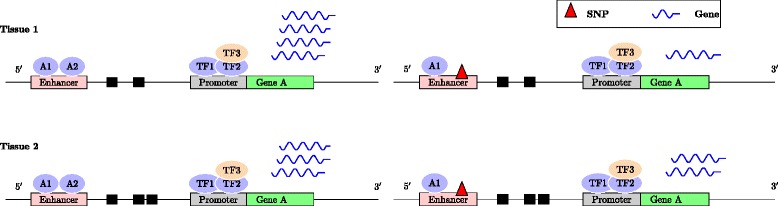
An illustration of the regulation of tissue-specific gene regulation. In this example, we illustrate the concept of tissue-specific gene expression using Gene A (quantified by *blue squiggly lines*) and its genetic variant (denoted by *red triangle* labeled SNP) in two tissues, tissue 1 and tissue 2. In both tissues 1 and 2, *left panel* indicates the wild-type gene expression of Gene A and the *right panel* indicates a reduced gene expression in the presence of a genetic variant, shown here by the reduced number of *blue squiggly lines*. It is clear from the figure that there is a difference in baseline gene expression levels of Gene A in tissues 1 and 2 and there is a difference in the degree to which the gene expression is repressed by the genetic variant

Combining genetic and gene expression data has emerged as a powerful strategy for systematically unraveling the effects of genetic variation on disease [1]. A common approach is to identify genetic variants that are correlated with gene expression in one or more genes [2]. Such variants are referred to as expression quantitative trait loci (eQTL). Since regulatory regions in higher eukaryotes activate gene transcription in a tissue-specific manner, genetic variants found within these regulatory regions may have variable effects on gene expression across different tissues or cell-types (Fig. 1). For example, a genetic variant found near the promoter region of the catechol-O-methyl transferase (*COMT*) gene, which has been implicated in schizophrenia, is associated with differential *COMT* expression across regions of the brain during the course of the illness [3]. This spatio-temporal gene expression pattern has been shown to be strongly associated with structural abnormalities such as the loss of brain volume in the frontal cortex and hippocampus that is part of the natural progression of schizophrenia [4]. Studying the underlying biology of this tissue- or region-specific gene expression variation is essential in understanding various complex diseases [2].

Many approaches to identifying eQTL utilize a marginal analysis of a single variant’s effect on gene expression in a single tissue. Such analyses are then repeated on each tissue leading to a tissue-by-tissue (TBT) approach [5–10]. However, such an approach has at least three significant limitations: First, a TBT analysis fails to fully exploit expression patterns across the tissues either by pooling information when a variant has a similar effect across multiple tissues or by explicitly identifying effects that differ across tissues. Second, marginal analyses of individual tissues lead to a proliferation of hypotheses tested, which can negatively impact the power of eQTL discovery. Third, even when one identifies a variant that affects expression of a given gene in a given tissue via a tissue-by-tissue approach, it is not clear whether the effect is tissue-specific or is shared across multiple tissues since such a hypothesis is not explicitly tested. Hence, multi-tissue eQTL studies, such as the Genotype-Tissue Expression (GTEx) project [11], in which expression is measured in up to 30 tissue sites in each individual, require new analytic approaches to fully exploit the information in these samples. Recently, two methods have been proposed that attempt to take better advantage of the information across multiple tissues. Sul et al. proposed the MetaTissue (MT) approach [12], which combines tissue-specific effects across multiple tissues in a meta-analytic framework. MT uses a mixed effects meta analytic framework that not only accounts for the correlation of gene expression between tissues but also heterogeneity of the effects across tissues. Flutre et al. proposed a Bayesian hierarchical model (eQTL-BMA) that models the joint distribution of gene expression across tissues and “combines information across genes to estimate the relative frequency of patterns of eQTL sharing among tissues” [13].

Both MT and eQTL-BMA require optimization under the alternative hypothesis (the given SNP is an eQTL in at least 1 tissue), and thus require the estimation of all model parameters for each gene by variant combination. As a result, the computational demands of both approaches scale very poorly with increasing numbers of variants or genes. To address this issue, we propose a score test-based approach which does not require parameter estimation under the alternative hypothesis. As a result, model parameters only have to be estimated once per genome, significantly decreasing computation time. Further, our score-based approach only requires estimation of the first two moments of the random effects, thus it is robust to misspecification of the random effect distribution [14]. We evaluate our method using extensive simulation studies that show a significant increase in power to detect eQTL when compared to a TBT approach. Furthermore, we show that our method surpasses currently existing joint modeling approaches such as MetaTissue eQTL and eQTL-BMA in terms of computational speed and yet provides a comparable performance with respect to statistical power. Finally, we demonstrate its effectiveness by applying it to two publicly available expression datasets from normal brains and show that by jointly analyzing multiple brain regions (tissues), we identify eQTL within more genes relative to a TBT analysis.

## Results and discussion

### Methods overview

For a given gene-SNP pair, our approach models gene expression across tissues using a linear mixed model in which both fixed and random effects are used to capture the effect of a variant on gene expression. Briefly, for each tissue *t* and individual *i* we model the potential genetic association between a target SNP and the expression levels of a target gene *j* at a single locus by using the following vectorized form of the linear-mixed model (the *t*-variate normal law with mean $\mu \in \mathbb {R}^{t}$ and variance $\Sigma \in \mathbb {R}^{t \times t}$ will be denoted as *N*_*t*_(*μ*,*Σ*)) –

1 $$ y_{ij} = \alpha_{j} + \mathbf{1} \beta_{j} g_{i} + \mathbf{1} u_{i} + g_{i} v_{j} + \xi_{ij} \qquad \xi_{ij} \overset{i.i.d.} \sim N_{t} \left(0, \epsilon \mathbb{I} \right)  $$

where *y*_*ij*_ is a *t*×1 vector of gene expression data, $\mathbb {I}$ denotes the corresponding *t*×*t* diagonal matrix, $\alpha \in \mathbb {R}^{t}$ is the fixed effect for the mRNA level for *t* tissues, *β*_*j*_ is the fixed effect for the SNP ($\beta _{j} \in \mathbb {R}^{1}$), *g*_*i*_ is the value of a bi-allelic genotype such that *g*_*i*_∈(0,1,2), which represents the number of copies of the minor allele. **1** denotes a column vector of *t* ones. The random effect $v_{j} \in \mathbb {R}^{t}$ represents tissue-specific interaction with the genotype and $u_{i} \in \mathbb {R}^{1}$ is a subject-specific random intercept. We assume that the random effects are independent and that $v_{j} \sim N_{t} \left (0, \gamma \mathbb {I} \right)$ and *u*_*i*_∼*N*_1_(0,*τ*).

Since tissue-specific effects are modeled as random effects, a test of whether there are tissue-specific effects is equivalent to testing whether the variance of the random effect (*γ*) is zero. Thus our approach involves testing only two scalar parameters (*β* and *γ*), regardless of the number of tissues being considered. We develop a score test of the null hypothesis that both of these parameters are zero, i.e., that the variant does not affect gene expression across any of the tissues. We present this model and the resulting score test in detail in the methods section.

### Simulations

We evaluate our approach through extensive simulation studies. We begin with a single locus and a single gene, of which the expression is measured across either 5 or 10 tissues. Genotypes are first generated assuming Hardy-Weinberg equilibrium and common minor allele frequency (>5 *%*). Given this genotype, gene expression is generated according to Eq. 1. Type I error is evaluated using 10,000 data replicates; 1,000 replicates are used for power calculations. Simulations under the null hypothesis confirm that our method has the correct type I error (Table 1).

**Table 1:** Table comparing the type I error of the joint score test statistic, *U*_*ψ*_ with tissue-by-tissue (TBT) analysis, MetaTissue (MT) model (FE = Fixed Effects model; RE = Random Effects model) and multivariate Bayesian Model Averaging. Note that all the results are based on 5,000 simulations on 100 observations at a nominal level of *α*=0.05

	Number of tissues = 5					Number of tissues = 10				
MAF	TBT	*M* *T*(*F* *E*)	MT(RE)	BMA	*U*_*ψ*_	TBT	*M* *T*(*F* *E*)	MT(RE)	BMA	*U*_*ψ*_
0.05	0.0422	0.0410	0.0488	0.0488	0.0456	0.0434	0.0362	0.0404	0.0392	0.0416
0.10	0.0476	0.0442	0.0510	0.0488	0.0494	0.0512	0.0368	0.0472	0.0448	0.0480

**Fig. 2:**
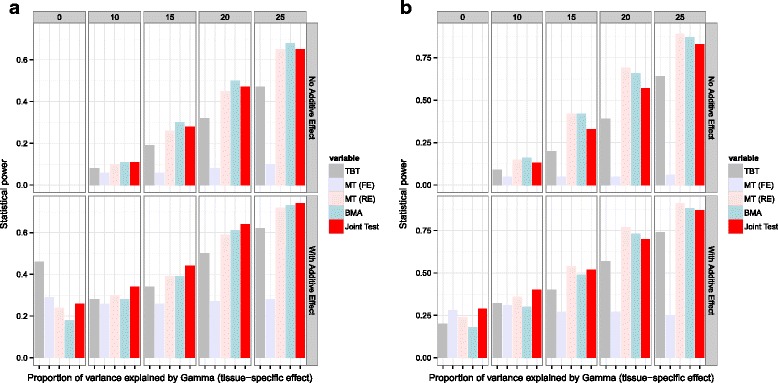
Statistical power comparison at a minor allele frequency of 0.05 (moderately rare variant minor allele frequency). Barplot depicting the statistical power comparison between the joint score test method and other methods such as MetaTissue model (Fixed Effects, FE; Random Effects, RE) and eQTL-BMA when the (**a**) number of tissues is 5 and (**b**) 10 at a minor allele frequency of 0.05. We varied the proportion of variance explained by *γ*(*P* *V* *E*_*γ*_) between 0 - 25 % and *β* fixed effect for the additive effect of the SNP. Each vertical grid labeled 0 through 25 represents varying levels of *P* *V* *E*_*γ*_ whereas each horizontal grid represents the presence or absence of the additive effect due to SNP

**Fig. 3:**
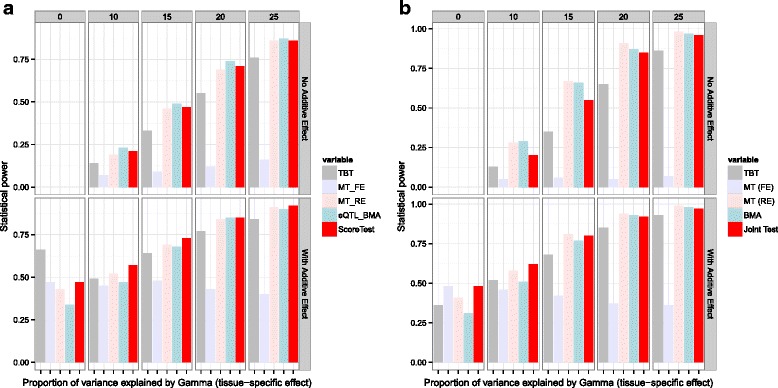
Statistical power comparison at a minor allele frequency of 0.1 (common variant minor allele frequency). Barplot depicting the statistical power comparison between the joint score test method and other methods such as MetaTissue model (Fixed Effects, FE; Random Effects, RE) and eQTL-BMA when the (**a**) number of tissues is 5 and (**b**) 10 at a minor allele frequency of 0.10. We varied the proportion of variance explained by *γ*(*P* *V* *E*_*γ*_) between 0 - 25 % and *β* fixed effect for the additive effect of the SNP. Each vertical grid labeled 0 through 25 represents varying levels of *P* *V* *E*_*γ*_ whereas each horizontal grid represents the presence or absence of the additive effect due to SNP

We compare the performance of our method with TBT, MT, and eQTL-BMA. Figure 2a shows that our method outperforms other methods in the presence of an additive genetic effect and tissue-specific interaction with the genotype (*PVE*_*γ*_) when the number of tissues is 5 at a moderately rare allele frequency of 0.05. When the number of tissues is increased to 10, MT seems to outperform all other methods, including ours as seen in Fig. 2b. On the other hand, at a more common variant frequency of 0.10 and when the number of tissues if 5 (Fig. 3a), our method outperforms eQTL-BMA and MT in the presence of both additive genetic effect and *PVE*_*γ*_. In the absence of any additive genetic effect, eQTL-BMA seems to work the best. However, when the number of tissues is increased to 10 (Fig. 3b) in the presence of any additive genetic effect, our method is comparable to MT and eQTL-BMA and better than TBT. The CPU time for the analyses performed on a simulated dataset are summarized in Table 2. It is important to note that these times are reflective of the algorithm and do not account for data pre-processing. It is clear from the table that our method is computationally faster than currently used multi-tissue eQTL methods, MT and eQTL-BMA. This computational efficiency is attributed to the existence of a closed-form solution to the distribution of our joint score test statistic, which can be written as a function of the number of genes and variants. It is important to note that the computational efficiency of MT and eQTL-BMA methods is estimated using publicly available software versions.

**Table 2:** Performance of different methods on a simulated dataset

Method	Number of tissues = 5	Number of tissues = 10	Core algorithm implementation
Joint score test	0.48 s (with no permutations)	0.7s (with no permutations)	RcppArmadillo
	45 s (with permutations)	72 s (with permutations)	
eQTL-BMA	176 s	244 s	C++
MetaTissue	157 s	822 s	Java

All the computations were performed on a single core of an Intel Xeon E5-2650 2.60GHz CPU. These times do not include any data preparation time and are reflective of the core algorithm alone

We demonstrate the effectiveness of our approach by applying it to two datasets in which gene expression data, measured across various regions in normal brains, is paired with genome-wide single nucleotide polymorphism data. These datasets have previously been analyzed using a region-by-region (i.e., tissue-by-tissue) approach. We consider two different types of analyses: One that focuses on SNPs that lie within 100 kilobase up- and down-stream of the transcription start site of a gene (referred to here as the *cis* candidate region), and another that focuses on a genome-wide analysis. Due to the computational burden of genome-wide analyses using eQTL-BMA and MT methods, we only apply our joint score test and the TBT approach and assessed their performance by comparing the total number of genome-wide gene-SNP pairs deemed statistically significant at a Bonferroni threshold.

### Region-specific analysis of normal adult human brains

Adult human brains have distinct expression patterns across each brain region [24], and understanding the genetic control of gene expression across the brain regions may further our understanding of brain diseases by identifying possible disease susceptibility regions. We hypothesize that our approach will identify more genes with eQTL than were previously identified using a TBT approach. The first brain dataset, originally analyzed by Gibbs [23], consists of four brain regions (cerebellum, CRBLM; frontal cortex, FCTX; pons, PONS; temporal cortex, TCTX) from 150 neuropathologically normal patients. Gene expression and genotype data were assayed on Illumina HumanRef-8 Expression BeadChips and Infinium HumanHap550 Beadchips, respectively. Genotype data was preprocessed to remove uncommon variants (minor allele frequency less than 0.05) and the population being analyzed is homogeneous with respect to patient ethnicity. After standard genotype and gene-expression preprocessing and quality control procedures (see methods), the resulting dataset consisted of 400,973 SNPs and 18,983 genes. We considered two different analyses - a *cis* analysis, which effectively restricts our analysis to only *cis-*SNPs that are 100 kilobase pairs up- and down-stream of the transcription start site, and a genome-wide analysis. A *cis* analysis reduces the search space of potential gene-SNP pairs to 511,458, thus reducing the total number of hypotheses being tested. A region-by-region *cis* analysis of Gibbs et al. neuropathologically normal human brain data yielded the following statistically significant results (i.e., passed the Bonferroni multiple testing threshold of $2.44 \times 10^{-8}=\frac {0.05}{\# \text {tissues} \times 511,458}$): 1,547 gene-*cis*SNP pairs in CRBLM, 1,609 gene-*cis*SNP pairs in FCTX, 1,148 gene-*cis*SNP pairs in PONS and 1,341 gene-*cis*SNP pairs in TCTX. After 10,000 permutations, a region-by-region analysis yielded 2,367 genes with at least one *cis*-eQTL while our approach identifies 3,913 genes with at least one *cis*-eQTL or approximately 65 % more genes. Of note, approximately 98 % of these genes are present in the list of genes identified by the TBT approach. For comparison, while eQTL-BMA approach identifies 2,919 genes (23 % more than the TBT approach with a 73 % gene-overlap), MT method identifies 3,743 genes (58 % more than the TBT approach with a 74 % gene-overlap) using its fixed-effects model and 3,843 genes (62 % more than the TBT approach with a 79 % gene-overlap)) using its random effects model. A genome-wide TBT analysis of the same data yielded the following statistically significant results (i.e., passed the Bonferroni multiple testing threshold of $1.64\times 10^{-12}=\frac {0.05}{\# \text {tissues} \times \# \text {genes} \times \# \text {SNPs }}$): 716 gene-SNP pairs in CRBLM, 779 gene-SNP pairs in FCTX, 473 gene-SNP pairs in PONS and 630 gene-SNP pairs in TCTX; 245 gene-SNP pairs are shared among all the brain regions with a total of 1,277 gene-SNP pairs being unique among all the regions of the brain. The smaller numbers are attributed to the more conservative Bonferroni threshold due to increased number of hypotheses tested. In contrast, our score test approach significantly (Bonferroni threshold = $6.57\times 10^{-12}=\frac {0.05}{\# \text {genes} \times \# \text {SNPs }}$) implicates 2,602 unique gene-SNP pairs more than twice the number identified by the TBT approach.

The second brain dataset, originally analyzed by Ramaswamy et al. [24], consists of ten brain regions (cerebellum, CRBLM; frontal cortex, FCTX; hippocampus, HIPP; medulla, MEDU; occipital cortex, OCTX; putamen, PUTM; substantia nigra, SNIG; temporal cortex, TCTX; thalamus, THAL; intralobular white matter, WHMT) from 134 neuropathologically normal patients. The gene expression data was assayed on Affymetrix Human Exon 1.0 ST Array, which has both exon-level and gene-level gene expression data. In order to simplify our analysis, we made use of gene-level expression data where the expression levels of each exon for each gene/transcript were aggregated using the Winsorized mean [25]. Genotype data were assayed on Illumina Infinium HumanHap550 v3 and were subjected to standard quality control preprocessing (see Methods). This dataset contains many samples with missing gene expression values however, the missing data does not affect parameter estimation or inference under our method. In fact, the likelihood of the observed data has the same form as that of the missing data (see Additional file 1: Supplementary methods). After standard genotype and gene-expression preprocessing and quality control procedures (see methods), there were 627,126 SNPs and 25,501 genes to be analyzed. *cis*-eQTL analysis of this data yielded 2,714 genes with at least one eQTL in a TBT analysis while our approach yielded 5,413 genes with at least one eQTL with a 94 % gene-overlap. For comparison, while eQTL-BMA approach identifies 5,316 genes, MT method identifies 4,984 and 5,176 genes with eQTL using its fixed-effects model and its random effects model, respectively. A genome-wide TBT analysis of the same data yielded 6,698 unique gene-SNP pairs after adjusting for multiple hypotheses (Bonferroni threshold = $3.13\times 10^{-13}=\frac {0.05}{\# \text {tissues} \times \# \text {genes} \times \# \text {SNPs }}$). As was observed with Gibbs et al. data, our approach again identifies substantially more eQTLs, significantly implicating 10,392 unique gene-SNP pairs.

## Methods

### Efficient score functions for *β* and *γ*

We begin with a linear mixed effects model that models expression patterns across tissues as a function of genotype. In a matrix notation, for a given gene-SNP pair

2 $$ Y = J \alpha + G\beta + Zu + Xv + \xi,  $$

where *Y* is a *nt*-dimensional matrix of expression levels in *t* tissues and *n* individuals, *α* is a fixed effect representing the tissue-specific intercepts, *G* is a *nt*-dimensional matrix of genotypes, *β* is a fixed effect of genotype across tissue, *u*∼*N*(0,*τZZ*^*T*^) is a *nt*-dimensional matrix of subject-specific random effect, *v*∼*N*(0,*γXX*^*T*^) is a *nt*-dimensional matrix of tissue-specific random effects, and *ξ*∼*N*(0,*εI*_*nt*_) and *I* is the identity matrix. The matrices *J*, *Z* and *X* are design matrices with *X* being a function of genotype. *J* is *nt*×*t* dimensional matrix denoting the design matrix for the tissue-specific intercepts. *Z* is *nt*×*nt* design matrix for the subject-specific intercepts. *X* is a *nt*×*t* design matrix of stacked genotypes. The parameters of interest are *β* and *γ*; *α*, *τ* and *ε* are nuisance parameters.

We test the null hypothesis that *H*_0_:*β*=*γ*=0, i.e. the variant does not affect gene expression across any of the tissues. To do so, we compute the efficient scores for *β* and *γ* by projecting off components correlated with the nuisance parameters. From Eq. 1, the log-likelihood function of Y conditional on the genotype is –

$$\begin{aligned} \ell \left(\beta,\theta \right) = c - \frac{1}{2} \log|\Sigma|-\frac{1}{2}\left(Y-J\alpha-G\beta\right)^{T} \Sigma^{-1} \left(Y-J\alpha-G\beta\right) \end{aligned} $$

where *θ* represents the vector of the variances of all the random effects in *Σ* and c is a constant. Alternatively, under Eq. 1 and normality, we have

$$Y \sim N(J\alpha + G\beta,\Sigma) \quad with \quad \Sigma = \epsilon I + \tau Z Z^{T} + \gamma X X^{T}. $$

The efficient scores evaluated under the null are given by

3 $$ U_{\beta} = \left(G - \bar{G} \right)^{T} \hat\Sigma_{n}^{-1} \left(Y-J\hat\alpha\right),  $$

and

4 $$ U_{\gamma} = \frac{1}{2} \left(Y - J\hat{\alpha}\right)^{T} \hat\Sigma_{n}^{-1} XX^{T} \hat\Sigma_{n}^{-1} \left(Y - J\hat{\alpha}\right),  $$

where $\hat \Sigma = \hat \tau Z Z^{T} + \hat \epsilon I$ and $\hat \tau $ along with $\hat \epsilon $ are the maximum likelihood estimators of *τ* and *ε* under the null.

Following Huang et al. [15], we propose a weighted sum of *U*_*β*_ and *U*_*γ*_ to arrive at our joint score test statistic, *U*_*ψ*_. Since *U*_*β*_ is linear in *Y* while *U*_*γ*_ is quadratic, we propose the following rule to combine them –

5 $$\begin{array}{@{}rcl@{}} \begin{array}{ll} U_{\psi} &\equiv {a_{\beta}} U_{\beta}^{2} + {a_{\gamma}} U_{\gamma} \\ &= \left(Y-J\hat\alpha\right)^{T} \hat{\Sigma}_{n}^{-1} \left[ a_{\beta} \left(G - \bar{G} \right) \left(G - \bar{G} \right)^{T} \right. \\ & \quad\left. + a_{\gamma} \left(\frac{1}{2} X X^{T} \right) {\vphantom{\left(G - \bar{G} \right)^{T}}}\right] \hat{\Sigma}_{n}^{-1} \left(Y-J\hat\alpha \right), \\ \end{array} \end{array} $$

where *a*_*β*_ and *a*_*γ*_ are scalar constants chosen to minimize the variance of *U*_*ψ*_ (see Additional file 1 for details). Under the null, *U*_*ψ*_ is distributed as a mixture of chi-square random variables. Several approximation and exact methods were proposed to obtain the distribution of *U*_*ψ*_ [16]. Here, we use the Satterthwaite method [17] to approximate the *p* values from a scaled *χ*^2^ distribution by matching the first two moments as $U_{\psi } \sim \kappa \chi ^{2}_{\nu }$ where $\kappa = \frac {2 Var(U_{\psi })}{E[U_{\psi }]}$ and $\nu = \frac {2 E[U_{\psi }]^{2}}{Var(U_{\psi })}$.

### Simulation studies

Each simulated dataset was comprised of data from a single locus and a single gene, whose expression is measured across either 5 or 10 tissues. The data are generated prospectively, i.e., first genotypes are generated (assuming Hardy-Weinberg equilibrium and >5 *%* minor allele frequency), then, given genotype, gene expression is generated according to Eq. 1 of methods. We use 10,000 data replicates when evaluating type I error and 1,000 for power calculations. Simulations were performed by varying two parameters, *β* (additive genetic effect) and the proportion of variation explained by the random effect of genotype (${PVE}_{\gamma } \equiv \frac {\gamma }{\tau + \gamma + \epsilon }$). Additive genetic effect in the simulations was controlled by varying *β* between 0 (indicates the absence of additive genetic effect) and 0.5 (indicates the presence of additive genetic effect), and the tissue-specific interaction effect was controlled by varying *γ* between 0 (indicating no tissue-specific effects) and 25 %. A linear mixed effects model was fit using the package *lme4* [18,19] in the statistical environment R (R Core Team). MT and eQTL-BMA methods were run on the simulated datasets as per their respective software instructions. We picked the default option for calculating the Bayes Factors and performed joint analysis with permutations while using eQTL-BMA method. The statistic computed by the eQTL-BMA approach is given a frequentist interpretation by translating the test statistic (computed by the eQTL-BMA model) into a *p* value for each gene by comparing the observed values with simulated values obtained under the null after permuting the sample labels. The significance of an association between a gene-SNP pair in a TBT analysis is assessed by the *p* value obtained using *lm* function in R. The test statistic is the minimum *p* value over the total number of tissues from linear regressions performed separately in each tissue for each gene-SNP pair. Statistical significance was determined at a nominal *p* value of 0.05 for all power simulations (in case of TBT analysis, it is $\frac {0.05}{k}$ where *k* is the number of tissues). In order to assess the computing times of various algorithms, we performed a series of simulations as noted in Flutre et al. [13], with five or ten tissues measured in 100 unrelated individuals. Each simulation consisted of 3,705 gene-SNP pairs, at least half of which were “null" (i.e. SNP was not an eQTL in any tissue) and the other half following an alternative hypothesis that the SNP was an eQTL in at least *k* tissues with *k* varying from 1 to 5 or 10. The genotypes at each SNP in each individual were simulated with minor allele frequency 30 % and assuming Hardy-Weinberg equilibrium. Gene expression data was generated for 100 genes and 1,036 SNPs (in *cis* with at least one gene) as was explained in Flutre et al.

### Preprocessing Gibbs J.R. et al. normal brain data

Gene expression on four brain regions including cerebellum (CRBLM), frontal cortex (FCTX), caudal pons (PONS) and temporal cortex (TCTX) and SNP datasets are publicly available (Gene Expression Omnibus (GEO) Accession Number: **GSE15745**; dbGAP Study Accession: **phs000249.v1.p1**). Genotyping was done on Infinium HumanHap550 Beadchips to assay genotypes for 561,466 SNPs, from the cerebellum tissue samples while gene expression profiling of 22,184 mRNA transcripts was performed using Illumina HumanRef-8 Expression BeadChips. The genotype data was recoded into a SNP matrix of values 0, 1 and 2 representing minor allele counts under the additive model. Samples with African (GSM394931 in CRBLM, GSM395081 in FCTX, GSM395226 in PONS and GSM395374 in TCTX) and Asian (GSM394121 in CRBLM, GSM394263 in FCTX, GSM394405 in PONS and GSM394566 in TCTX) ancestry were removed from the analysis. These SNPs were filtered on the missing*ness* of the individual data (excluded samples with more than 10 % missing genotypes) and the SNP data (excluded SNPs with missing values), followed by MAF (included SNPs with MAF ≥0.05) and Hardy-Weinberg equilibrium (HWE; p-values <0.001) in the same order using PLINK [20] software. Top principal components on the filtered and pruned genotype data based on linkage disequilibrium measurements (window size of 1500, sliding window 150 SNPs at a time and an *r*^2^ threshold of 0.04) were generated using EIGENSTRAT [21] method for later use to correct for population stratification. Each gene expression probe was adjusted for the biological and methodological covariates such as tissue bank, gender, hybridization batch and numeric covariates such as post-mortem interval (PMI) and age in order to remove any associated confounding effects.

### Preprocessing Ramaswamy et al. normal brain data (BrainEAC consortium study)

Gene expression data from 10 brain regions including cerebellar cortex (CRBLM), frontal cortex (FCTX), hippocampus (HIPP), inferior olivary nucleus/medulla (MEDU), occipital cortex (OCTX), putamen (PUTM), substantia nigra (SNIG), temporal cortex (TCTX), thalamus (THAL) and intralobular white matter (WHMT) was obtained from GEO under the accession id **GSE46706**. The authors have kindly provided us with the SNP data. This data is part of the UK Brain Expression Consortium (UKBEC) and the brain samples were collected by the Medical Research Council Sudden Death Brain and Tissue Bank, Edinburgh, UK, and the Sun Health Research Institute an affiliate of Sun Health Corporation, USA. Exon-specific RNA expression was quantified using Affymetrix Human Exon 1.0 ST arrays and the genotyping was done on Illumina Omni1-quad and Immunochip arrays. We followed the same steps to preprocess the SNP data. Preprocessed gene-level expression profile was obtained as a ‘Series Matrix File’ from GEO where the gene-level expression data for every gene is aggregated over all the probes representing it. Gene-level summary signals were then generated by calculating the Winsorized mean of expression values of all probe sets annotated to a transcript. There are a total of 25,501 genes represented on this microarray platform.

It is important to note that our data preprocessing methods for both the aforementioned datasets are different from the original methods used by the authors in their respective publications.

### Data analysis

We performed two different types of data analyses - 1) one that focuses on *cis* candidate regions that are defined by the proximity of an eQTL to the transcription start site of a gene not exceeding 100 kilobase up- and down-stream of the transcription start site of a gene, and 2) a genome-wide analysis, which tests all gene-SNP pairs in a given eQTL dataset.

The performance of all the methods for the *cis* analysis is assessed by comparing the number of genes identified as having at least one eQTL in any given tissue at a 5 % false discovery rate (FDR). eQTL-BMA computes a test statistic for all genes as an average of all Bayes Factors for the given gene and its *cis*-SNP. This test statistic is then converted to a *p* value for each gene using an adaptive permutation-resampling technique performed on each gene separately, which compares the observed test statistic with the value of the test statistic obtained from repeated permutations (10,000 in this case). The MT model was run on the same set of *cis* SNPs for all the genes and the resulting *p* values using both fixed and random effects model were adjusted using the Benjamini-Hochberg method [22]. Since the MT model outputs *p* values for each gene-SNP pair being tested, we identified the genes with at least one eQTL by finding the minimum adjusted *p* value for a given gene. In the case of a TBT analysis, which tests for the presence of an eQTL in a single tissue, the test statistic is the minimum *p* value of the linear regressions between a gene and a candidate *cis*-SNP in that tissue. While applying our joint score test approach for every gene, we computed the minimum *p* value across all the candidate *cis*-SNPs and the adjusted *p* value for that gene was computed as the average number of times the permuted *p* values are smaller than the observed minimum *p* value.

Genome-wide analysis of the brain datasets was performed only using our joint score test and TBT approaches due to the computational burden such analyses create on MT and eQTL-BMA methods. The performance of both TBT and our method for the genome-wide analysis of the datasets was evaluated at Bonferroni thresholds by comparing the number of significant gene-SNP pairs. A generic TBT approach on the normal human brain datasets was implemented as a linear regression model in the R-package MatrixEQTL [5].

## Conclusion

Our method investigates the presence of two types of genetic effects: 1) an overall shift in gene expression due to genotype across all tissues, and 2) tissue-specific effects of genotypes on gene expression. Our approach models tissue-specific effects as random effects, resulting in a test that involves far fewer parameters. Further, our simulations demonstrate improved power over previously proposed methods for eQTL analysis.

Both of the datasets examined here used genome-wide association study (GWAS) SNP array platforms to interrogate germ-line variation. By design, the SNPs on these platforms are overwhelmingly common, and as a result, individual SNP based analyses will have reasonable power to detect association with gene expression. However, as eQTL studies transition from GWAS platforms to whole genome sequencing as the primary approach to assaying genetic variation, rare variation will also need to be considered. In this setting, an individual-variant analysis is no longer viable. Thus the dominant paradigm for rare variant analyses takes a gene-based or regional approach, accumulating information across a gene or other genetic unit. Score statistics are often used in this context and have been leveraged to form both burden- [26] and kernel-based [27] tests. The score test based framework presented here could be used in a similar way to develop tests that accumulates rare variant contributions across genomic regions that are annotated to have regulatory potential.

Another important issue in the context of eQTL studies is the vast number of hypotheses being tested. Because of this, it is important that multiple testing corrections are used to appropriately control for either family-wise error rate (FWER) or false discovery rate (FDR). Bonferroni (FWER) and Bejamini and Hochberg (FDR) adjustments are simple approaches to maintaining such control but they can be overly conservative when there is substantial correlation among the tests, which is likely in eQTL studies. Permutation based adjustment can address this limitation but may be computationally prohibitive. Resampling-based [15] and Monte Carlo [28] approaches have been proposed that allow the characterization of the permutation distribution of the statistics without the computational demands required of permutation. Investigating how these approaches can be adapted to the joint score test presented here is a topic for future research.

We are currently considering a couple of ways our model can be extended to incorporate additional data types. First, our approach could accommodate the analysis of RNA-Seq by modeling gene transcripts in an analogous fashion to tissues in our current formulation. Thus, one would be able to test for both a variant’s overall effect across all isoforms of a gene as well as transcript specific effects. Second, since DNA methylation can impact gene expression patterns in a tissue specific manner, we are considering an extension of the model that explicitly incorporates methylation in modeling the effect germ-line variation has on gene expression patterns.

## Abbreviations

BMA, Bayesian model averaging; eQTL, expression quantitative trait loci; FDR, false discovery rate; FWER, family wise error rate; GWAS, genome wide association study; MT, MetaTissue; SNP, single nucleotide polymorphism; TBT, tissue-by-tissue

## Additional file

Additional file 1 Supplementary text. This is a PDF document, which elaborates some methodological aspects of our paper. (PDF 200 kb)
