# Supplementary material for: Exploiting expression patterns across multiple tissues to map expression quantitative trait loci
Source: BMC Bioinformatics. 2016 Jun 24;17:257. doi: 10.1186/s12859-016-1123-5 (PMC4919894; doi:10.1186/s12859-016-1123-5)
Supplement: Additional file 1 — Supplementary text. This is a PDF document, which elaborates some methodological aspects of our paper. (PDF 200 kb) [file 12859_2016_1123_MOESM1_ESM.pdf]

Supplementary text for "Exploiting expression patterns across multiple tissues to map expression quantitative trait loci"

1 Our model

For a given gene-SNP pair, we begin with a linear mixed effects model that models expression patterns across tissues as a function of genotype, i.e.,

$$\mathbf{Y} = J\alpha + G\beta + Zu + Xv + \xi \tag{1}$$

where Y is a nt-dimensional vector of expression levels in t tissues and n individuals, α is a vector of tissue-specific intercepts, G is a nt-dimensional vector of genotypes, β is a fixed effect of genotype across tissue, $u \sim N\left(0, \tau ZZ^T\right)$ is a vector of subject-specific random effect, $v \sim N\left(0, \gamma XX^T\right)$ is a vector of tissue-specific random effects, and $\xi \sim N\left(0, \epsilon I_{nt}\right)$. The matrices J, Z and X are design matrices with X being a function of genotype. J is $nt \times t$ dimensional matrix denoting the design matrix for the tissue-specific intercepts. Z is $nt \times nt$ design matrix for the subject-specific intercepts. X is a $nt \times t$ design matrix of stacked genotypes. The parameters of interest are β and γ; α, τ and ϵ are nuisance parameters.

We test the null hypothesis that $H_0 : \beta = \gamma = 0$, i.e. the variant does not affect gene expression across any of the tissues.

2 Derivation

From equation 1, the log-likelihood function of Y conditioned on the genotype is –

$$\ell\left(\beta, \theta\right) = c - \frac{1}{2}\log|\Sigma| - \frac{1}{2}\left(Y - J\alpha - G\beta\right)^T \Sigma^{-1}\left(Y - J\alpha - G\beta\right) \tag{2}$$

where θ represents the vector of all the variance components involved in Σ and c is a constant. Alternatively, under equation 1 and normality, we have

$$Y \sim N(J\alpha + G\beta, \Sigma) \quad with \quad \Sigma = \epsilon I + \tau ZZ^T + \gamma XX^T$$

From Jiming Jiang's *Linear and Generalized Linear Mixed Models and Their Applications* [1] –

$$\frac{\partial \ell}{\partial \beta} = G^T \Sigma^{-1} Y - G^T \Sigma^{-1} G \beta$$

$$\frac{\partial \ell}{\partial \theta_r} = \frac{1}{2} \left\{ (Y - G\beta - J\alpha)^T \Sigma^{-1} \frac{\partial \Sigma}{\partial \theta_r} \Sigma^{-1} (Y - G\beta - J\alpha) - \text{Tr}\left(\Sigma^{-1} \frac{\partial \Sigma}{\partial \theta_r} \right) \right\}$$

where θ_r is the rth component of θ such that $\theta \in (\tau, \gamma, \epsilon)$.

$$E\left[\frac{\partial^2 \ell_i}{\partial \beta \partial \beta^T} \right] = -G^T \Sigma^{-1} G$$

$$E\left[\frac{\partial^2 \ell_i}{\partial \beta \partial \theta_r} \right] = 0$$

$$E\left[\frac{\partial^2 \ell_i}{\partial \theta_r \partial \theta_s} \right] = -\frac{1}{2} \text{Tr}\left(\Sigma^{-1} \frac{\partial \Sigma}{\partial \theta_r} \Sigma^{-1} \frac{\partial \Sigma}{\partial \theta_s} \right)$$

2.1 Score test

Let the <u>parameters of interest</u> be $\psi = (\beta, \gamma)^T$ and the <u>nuisance parameters</u> be $\eta = (\alpha, \tau, \epsilon)^T$. The following is constructed under the null (H_0)

$$U_\psi = \begin{pmatrix} \frac{\partial l}{\partial \beta} \\ \frac{\partial l}{\partial \gamma} \end{pmatrix} - \begin{bmatrix} I_{\beta\alpha} & I_{\beta\tau} & I_{\beta\epsilon} \\ I_{\gamma\alpha} & I_{\gamma\tau} & I_{\gamma\epsilon} \end{bmatrix} \begin{bmatrix} I_{\alpha\alpha} & I_{\alpha\tau} & I_{\alpha\epsilon} \\ I_{\tau\alpha} & I_{\tau\tau} & I_{\tau\epsilon} \\ I_{\epsilon\alpha} & I_{\epsilon\tau} & I_{\epsilon\epsilon} \end{bmatrix}^{-1} \begin{bmatrix} \frac{\partial l}{\partial \alpha} \\ \frac{\partial l}{\partial \tau} \\ \frac{\partial l}{\partial \epsilon} \end{bmatrix}$$

Some algebra will result in the following –

$$U_\beta = \left(G - \bar{G} \right)^T \hat{\Sigma}_n^{-1} (Y - J\hat{\alpha}) \tag{3}$$

and

$$U_\gamma = \frac{1}{2} (Y - J\hat{\alpha})^T \hat{\Sigma}_n^{-1} X X^T \hat{\Sigma}_n^{-1} (Y - J\hat{\alpha}) \tag{4}$$

$$\begin{aligned} U_\psi &= \left(\boldsymbol{a_\beta} U_\beta^2 + \boldsymbol{a_\gamma} U_\gamma \right) \\ &= (Y - J\hat{\alpha})^T \hat{\Sigma}_n^{-1} \left[a_\beta \left(G - \bar{G} \right) \left(G - \bar{G} \right)^T + a_\gamma \frac{1}{2} X X^T \right] \hat{\Sigma}_n^{-1} (Y - J\hat{\alpha}) \end{aligned} \tag{5}$$

2.2 Missing response data

Let $Y_i = \{Y_i^o, Y_i^m\}$ with Y_i^o the observed part and Y_i^m the missing part. Also, let $R_{i,j} = 1$ if $Y_{i,j}$ is observed and $R_{i,j} = 0$ otherwise. Assume that all the explanatory variables are completely observed. θ and ψ describe the measurement and missingness, respectively.

$$f\left(Y^o, R|\theta,\psi\right) = \int f\left(Y^o, Y^m|\theta\right) f\left(R|Y^o, Y^m,\psi\right) dY^m$$

Assuming that the data are missing at random (MAR),

$$\begin{aligned} f\left(Y^o, R|\theta,\psi\right) &= \int f\left(Y^o, Y^m|\theta\right) f\left(R|Y^o,\psi\right) dY^m \\ &= f\left(R|Y^o, Y^m,\psi\right) \int f\left(Y^o, Y^m|\theta\right) dY^m \\ &= f\left(R|Y^o,\psi\right) f\left(Y^o|\theta\right) \end{aligned}$$

If the parameter space of $(\theta', \psi')'$ is the product of the parameter spaces of θ and ψ (separability condition), then the inference is based on the observable data only (ignorability) [3, 2].

If $x = [x_1, x_2]$ and $x \sim N(x, \mu, \Sigma)$ and x_1 constitute gene expression data available for samples with all the tissues/groups while x_2 constitutes gene expression data for samples with depleted tissues/groups. The multivariate gaussian theorem states that the marginal distribution of x_1 and x_2 are also normal with mean vector μ_i and covariance matrix Σ_{ii} $(i = 1, 2)$, respectively. The conditional distribution of x_i given x_j is also normal with mean vector such that $\mu_{i|j} = \mu_i + \Sigma_{ij}\Sigma_{ij}^{-1}(x_j - \mu_j)$ and $\Sigma_{i|j} = \Sigma_{jj} - \Sigma_{ij}^T\Sigma_{ij}^{-1}\Sigma_{ij}$.

The joint density of x is given by

$$L_n\left(x_1, x_2\right) = \prod_{i=1}^{n} (2\pi)^{-\frac{n}{2}} \mid \Sigma_i \mid^{-\frac{1}{2}} exp\left[-\frac{1}{2}Q\left(x_1, x_2\right)\right]$$

where

$$Q\left(x_1, x_2\right) = (x-\mu)^T \Sigma^{-1} (x-\mu)$$

After some algebra, we can show that the marginal distribution of x_1 can be written as

$$f_1\left(x_1\right) = \int f\left(x_1, x_2\right) dx_2 = \frac{1}{(2\pi)^{\frac{p}{2}} \mid \Sigma_{11} \mid^{\frac{1}{2}}} exp\left[-\frac{1}{2}\left(x_1-\mu_1\right)^T \Sigma_{11}^{-1}\left(x_1-\mu_1\right)\right]$$

...and the conditional distribution of x_2 given x_1 is given by

$$
\begin{aligned}
f_{2|1}\left(x_1, x_2\right) &= \frac{f\left(x_1, x_2\right)}{f\left(x_1\right)} \\
&= \frac{1}{(2\pi)^{\frac{q}{2}} \mid A \mid^{\frac{1}{2}}} exp\left[-\frac{1}{2}\left(x_2 - b\right)^T A^{-1}\left(x_2 - b\right)\right]
\end{aligned}
$$

where $b = \mu_2 + \Sigma_{12}^T \Sigma_{11}^{-1}\left(x_1 - \mu_1\right)$ and $A = \Sigma_{22} - \Sigma_{12}^T \Sigma_{11}^{-1} \Sigma_{12}$.

In this way, we can show that the observed data likelihood has the exact same model form as the full data likelihood.

3 Variance-covariance of U_β^2 and U_γ

We have

$$
\mathbf{Y} = J\alpha + G\beta + Zu + Xv + \xi \qquad Y \sim N(J\alpha + G\beta, \Sigma) \tag{6}
$$

From section 2.1, at global null i.e. $H_0 : \beta = 0; \gamma = 0$, we have

$$
U_\gamma = \frac{1}{2} Y^T \Sigma_n^{-1} X X^T \Sigma_n^{-1} Y \tag{7}
$$

where $\Sigma = \hat{\epsilon} I + \hat{\tau} Z Z^T$, $\hat{\tau}$ and $\hat{\epsilon}$ are the maximum likelihood estimates of the individual-specific and tissue-specific random effects. Using the theory of quadratic forms [1], estimated variance of U_γ under the null is given by

$$
Var_{H_0}\left(U_\gamma\right) = 2\,\mathrm{Tr}\left[\left(\Sigma^{-1}\frac{1}{2} X X^T \Sigma^{-1}\right)\Sigma\left(\Sigma^{-1}\frac{1}{2} X X^T \Sigma^{-1}\right)\Sigma\right] \tag{8}
$$

Similarly, from section 2.1,

$$
U_\beta^2 = Y^T \Sigma^{-1}\left(G - \bar{G}\right)\left(G - \bar{G}\right)^T \Sigma^{-1} Y \tag{9}
$$

From the theory of quadratic forms [1], estimated variance of U_β^2 under the null is

$$
Var_{H_0}\left(U_\beta^2\right) = 2\,\mathrm{Tr}\left[\left(\Sigma^{-1}\left(G - \bar{G}\right)\left(G - \bar{G}\right)^T \Sigma^{-1}\right)\Sigma\left(\Sigma^{-1}\left(G - \bar{G}\right)\left(G - \bar{G}\right)^T \Sigma^{-1}\right)\Sigma\right] \tag{10}
$$

U_β^2 and U_γ share the same ϵ. Again, from the theory of the quadratic forms, the covariance between U_β^2 and U_γ is

$$
Cov_{H_0}\left(U_\beta^2, U_\gamma\right) = 2\,\mathrm{Tr}\left[\left(\Sigma^{-1}\left(G - \bar{G}\right)\left(G - \bar{G}\right)^T \Sigma^{-1}\right)\Sigma\left(\Sigma^{-1}\frac{1}{2} X X^T \Sigma^{-1}\right)\Sigma\right] \tag{11}
$$

3.1 Optimal weights for minimum variance linear combination

Let $a = (a_\beta, a_\gamma)^T$, $U_\psi = \left(U_\beta^2, U_\gamma\right)$, and $V_\psi = Var\left(U_\psi\right)$. We want to find the minimum variance linear combination $a^T V_\psi$, subject to the constraint that $a_\beta + a_\gamma = 1$ or $a^T\mathbf{1} = 1$. Specifically, we wish to minimize $a^T V_\psi a$.

Using Lagrangian multipliers to perform constrained optimization, we see that

$$\mathcal{L}\left(a|\lambda\right) = a^T V_\psi a - \lambda\left(a^T\mathbf{1} - 1\right)$$

where $\mathbf{1} = \begin{bmatrix}1 & 1\end{bmatrix}^T$ and $\lambda > 0$.

$$\frac{\partial}{\partial\left(a^T, \lambda\right)} = \left(a^T V_\psi a - \lambda\left(a^T\mathbf{1} - 1\right)\right) = 0$$

From the above equations, we have the following system of equations–

$$2V_\psi a - \lambda\mathbf{1} = 0 \qquad\qquad a^T\mathbf{1} = \mathbf{1}^T a = 1$$

$$a = \frac{\lambda}{2} V_\psi^{-1}\mathbf{1}$$

and

$$1 = a\mathbf{1}^T = \frac{\lambda}{2}\mathbf{1}^T V_\psi^{-1}\mathbf{1}$$

so that,

$$\lambda = \frac{2}{\mathbf{1}^T V_\psi^{-1}\mathbf{1}}$$

This gives our optimal weights –

$$a = \frac{V_\psi^{-1}\mathbf{1}}{\mathbf{1}^T V_\psi^{-1}\mathbf{1}}$$

$$a_\gamma = \frac{var\left(U_\beta^2\right) - cov\left(U_\beta^2, U_\gamma\right)}{var\left(U_\beta^2\right) + var\left(U_\gamma\right) - 2cov\left(U_\beta^2, U_\gamma\right)} \tag{12}$$

and

$$a_\beta = \frac{var\left(U_\gamma\right) - cov\left(U_\beta^2, U_\gamma\right)}{var\left(U_\beta^2\right) + var\left(U_\gamma\right) - 2cov\left(U_\beta^2, U_\gamma\right)} \tag{13}$$

4 MetaTissue method

MetaTissue (MT) method was proposed by Sul *et al* that jointly models all tissues by utilizing a meta-analysis by extending it to a mixed-model framework. A mixed model is used to account for the correlation of expression between tissues, and perform meta-analysis to combine results from multiple tissues. The following model description is from the original paper.

Consider the following mixed-model –

$$Y = 1\alpha + X_j\beta + u + e$$

where $u \sim N\left(0, \sigma_\mu^2 D\right)$ and $e \sim (0, \sigma_e^2 I)$ and X_j is the matrix denoting SNP j for T tissues. The variances are estimated using *EMMA* and $\hat{\beta}$s are jointly estimated using the following equation –

$$\hat{\beta} = \left(X_j' \Sigma^{-1} X_j\right)^{-1} X_j' \Sigma^{-1} Y$$

Given the $\hat{\beta} = \left(\hat{\beta}_1, \ldots, \hat{\beta}_T\right)$, information from multiple tissues is combined by applying meta-analysis to $\hat{\beta}$.

4.1 Fixed-effects model

A statistic of FE and its distribution under the null hypothesis are –

$$S_{FE} = \frac{\sum_{i=1}^{T} V_i^{-1} B_i}{\sqrt{\sum_{i=1}^{T} V_i^{-1}}} \sim N(0, 1)$$

where $B_1 \ldots B_T$ and $V_1, \ldots, V_T$ are the estimates of effect-size and the standard error of B_i in T tissues. Let μ be the unknown true effect size and so the null hypothesis of FE is $\mu = 0$ or in other words the effect size in all tissues is zero. A p-value of S_{FE} is obtained from the standard normal distribution.

Under the null hypothesis, $\frac{\hat{\beta}}{\sqrt{var(\hat{\beta})}}$ will approximately follow *t-distribution* with k degrees of freedom.

$$p_t = 2\left(1 - \phi_{t(k)}\left(\frac{\mid \hat{\beta} \mid}{\sqrt{var\left(\hat{\beta}\right)}}\right)\right)$$

4.2 Random-effects model

The general assumption behind the random-effects model is that the effect size of a variant is different among datasets and follows a probability distribution with mean μ and variance τ^2. The H_0 is the same as that of the fixed-effects model – $H_0 : \mu = 0$. The statistic for the random effects model is defined as –

$$S_{RE} = \sum log\left(\frac{V_i}{V_i + \hat{\tau^2}}\right) + \sum \frac{B_i^2}{V_i} - \sum \frac{(B_i - \hat{\mu})^2}{V_i + \hat{\tau^2}}$$

where $\hat{\mu}$ and $\hat{\tau^2}$ are estimated mean and variance of the effect size, and the *maximum likelihood estimates* of the two parameters that are iteratively calculated using *Hardy and Thompson approach* or some other iterative approach. The statistic follows a half and half mixture of χ_0^2 and χ_1^2 under the null.

5 eQTL-BMA method

eQTL-BMA, proposed by Flutre *et al*, investigates whether the SNP is an eQTL in any tissue, and, if so, in which tissues. The primary model is

$$y_{si} = \mu_s + \beta_s g_i + \epsilon_{si} \qquad \epsilon_{si} \sim N\left(0, \sigma_s^2\right)$$

where y_{si} denotes gene expression vector in tissue s, for i^{th} individual, μ_s is the mean expression level of the gene in tissue s, β_s is the effect of the gene on the genotype in tissue s and g_i is the genotype of individual i coded as 0,1 or 2 copies of the reference allele. Statistical inference is made on γ, a binary variable (called configuration) whose status indicates the presence or absence of an eQTL. The length of γ depends on the number of tissues. Null hypothesis is indicated by $\gamma = \{0, \ldots, 0\}$ and any other combination is considered an alternative hypothesis. The statistical inference on γ is done using Bayes Factors such that –

$$BF_\gamma = \frac{P\left(data|\gamma\right)}{P\left(data|H_0\right)}$$

In order to account for many possible alternatives, the overall strength of evidence against at the candidate SNP is obtained by "Bayesian Model Averaging" (BMA), which involves averaging over the possible alternative configurations, weighting each by its prior probability, $\eta_\gamma = P\left(\gamma|H_0 = FALSE\right)$:

$$BF_{BMA} = \frac{P\left(data|H_0 = false\right)}{P\left(data|H_0 = true\right)} = \sum_{\gamma \neq 0} \eta_\gamma BF_\gamma$$

Large values of BF_{BMA} indicate a strong evidence against the H_0. Another flavor BF_{BMA}^{HM} indicates a hierarchical model where the hyperparameters are estimated from the data (data-driven approach). $BF_{BMAlite}$ is a more computationally scalable version of the above flavors as

it averages the test statistic over $S+1$ configurations. In general, eQTL-BMA method does not scale well with increasing number of tissues because the number of terms in the sum of above equation is $2^S - 1$.

In the presence of a strong evidence against the H_0, posterior probability on each configuration indicating that the SNP is an eQTL in tissue s is computed by –

$$P\left(\gamma = TRUE|data, H_0 = false\right) = \frac{\eta_\gamma BF_\gamma}{\sum\limits_{\gamma=0} \eta_\gamma BF_\gamma}$$

A frequentist interpretation to Bayes Factors computed by eQTL-BMA is given by performing adaptive permutations at the gene-level at a given FDR.

6 A note on statistical software

Our simulations were run to compare the statistical power (and type I error rate) between our method, eQTL-BMA, MetaTissue and Tissue-by-Tissue methods.

eQTL-BMA software is available for download at `https://github.com/timflutre/eqtlbma`. In order to expedite the analysis, we ran $BF_{BMAlite}$ version of the software, 1,000 adaptive permutations (using trick 1) to obtain the gene-level p value. These p values were then extracted from *output.log˙jointPermPvals.txt.gz* file for further analysis. We used eQTL-BMA software version 1.2 to perform all the analyses. In case of the real data analyses, we increased the number of permutations to the author recommended 10,000.

MetaTissue model software is made available at `http://genetics.cs.ucla.edu/metatissue/`. We used default setting for each step described by the author on the website. We used MetaTissue software version 0.3 for our analyses.

References

[1] J Jiang. *Linear and Generalized Linear Mixed Models and Their Applications.* Springer Series in Statistics. Springer-Verlag New York, 233 Springer Street, New York, NY 10013, USA, 1 edition, 2007.

[2] R.J. Little and D.B. Rubin. *Statistical Analysis with Missing Data.* John Wiley and Sons, 111 River Street, Hoboken, NJ 07030, USA, 2 edition, 2002.

[3] G Verbeke and G Molenberghs. *Linear Mixed Models for Longitudinal Data.* Springer-Verlag New York, 175 Fifth Avenue, New York NY 10010, USA, 1 edition, 2000.
